# Prognostic Value of the Modified Rutgeerts Score for Long-Term Outcomes After Primary Ileocecal Resection in Crohn's Disease

**DOI:** 10.14309/ajg.0000000000002509

**Published:** 2023-11-01

**Authors:** Michiel T.J. Bak, Sebastiaan ten Bokkel Huinink, Nicole S. Erler, Alexander G.L. Bodelier, Gerard Dijkstra, Mariëlle Romberg-Camps, Nanne K.H. de Boer, Frank Hoentjen, Laurents P.S. Stassen, Andrea E. van der Meulen–de Jong, Rachel L. West, Oddeke van Ruler, C. Janneke van der Woude, Annemarie C. de Vries

**Affiliations:** 1 Department of Gastroenterology and Hepatology, Erasmus University Medical Center Rotterdam, Rotterdam, the Netherlands;; 2 Department of Epidemiology, Erasmus MC University Medical Center, Rotterdam, the Netherlands;; 3 Department of Biostatistics, Erasmus MC University Medical Center, Rotterdam, the Netherlands;; 4 Department of Gastroenterology and Hepatology, Amphia Hospital, Breda, the Netherlands;; 5 Department of Gastroenterology and Hepatology, University Medical Center Groningen, Groningen, the Netherlands;; 6 Department of Gastroenterology, Geriatrics, Internal and Intensive Care Medicine (Co-MIK), Zuyderland Medical Centre, Heerlen-Sittard-Geleen, the Netherlands;; 7 Department of Gastroenterology and Hepatology, Amsterdam University Medical Center, Amsterdam, the Netherlands;; 8 Department of Gastroenterology and Hepatology, Radboud University Medical Center, Nijmegen, the Netherlands;; 9 Division of Gastroenterology, University of Alberta, Edmonton, Canada;; 10 Department of Surgery, Maastricht University Medical Center, Maastricht, the Netherlands;; 11 Department of Gastroenterology and Hepatology, Leiden University Medical Center, Leiden, the Netherlands;; 12 Department of Gastroenterology and Hepatology, Franciscus Gasthuis & Vlietland, Rotterdam, the Netherlands;; 13 Department of Surgery, IJsselland Ziekenhuis, Cappelle aan den IJssel, the Netherlands;; 14 Department of Surgery, Erasmus University Medical Center Rotterdam, Rotterdam, the Netherlands.

**Keywords:** Crohn's disease, modified Rutgeerts score, postoperative recurrence

## Abstract

**INTRODUCTION:**

The prognostic value of the modified Rutgeerts score (mRS) in patients with Crohn's disease (CD) needs to be further elucidated. This study assessed the prognostic value of the mRS for long-term outcomes after primary ileocecal resection in patients with CD.

**METHODS:**

Patients with CD after primary ileocecal resection with an available mRS at first postoperative ileocolonoscopy (index mRS) were retrospectively included. The primary outcome was surgical recurrence. Secondary outcomes were clinical recurrence and progression to severe endoscopic recurrence (≥i3). Cox proportional hazard models were used to assess the association between index mRS and outcomes.

**RESULTS:**

Six hundred fifty-two patients were included (mean follow-up: 6.4 years, SD: 4.6). Surgical recurrence rates were 7.7%, 5.3%, 12.9%, 19.1%, 28.8%, 47.8% for index mRS i0, i1, i2a, i2b, i3, and i4, respectively. Clinical recurrence occurred in 42.2% (i0), 53.7% (i1), 58.5% (i2a), 80.2% (i2b), 79.4% (i3), and 95.3% (i4) of patients. Progression to severe endoscopic recurrence occurred in 21.1% (i0), 33.9% (i1), 26.8% (i2a), and 33.3% (i2b) of patients. An index mRS of i2b (adjusted hazard ratio [aHR] 3.0; 1.5–5.6), i3 (aHR 4.0; 2.0–7.9) and i4 (aHR 8.0; 4.0–16.0) were associated with surgical recurrence. An index mRS of i1 (aHR 1.7; 1.2–2.4), i2a (aHR 1.7; 1.2–2.4), i2b (aHR 4.4; 3.2–6.0), i3 (aHR 3.6; 2.5–5.2), and i4 (aHR 7.3; 4.8–10.9) were associated with clinical recurrence. An index mRS of i1 (aHR 2.0; 1.1–3.7) or i2b (aHR 2.5; 1.4–4.6) was associated with progression to severe endoscopic recurrence.

**DISCUSSION:**

The increasing mRS corresponds closely with the risk of surgical and clinical recurrence. An index mRS ≥ i2b is associated with surgical recurrence, an index mRS ≥ i1 is associated with clinical recurrence, and i1 or i2b with progression to severe endoscopic recurrence. These results support tight monitoring of disease activity and treatment optimization in patients with ileal lesions and a more conservative management in patients with anastomotic lesions.

## Graphical abstract

**Figure:**
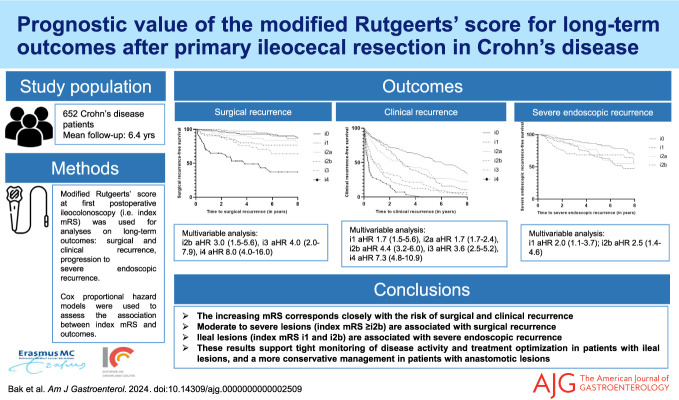

## INTRODUCTION

Patients with Crohn's disease (CD) are still at considerable risk of an intestinal resection although the risk has declined over the past decades (1). An intestinal resection is an important treatment modality, which is performed in approximately 25% of patients within 10 years after CD diagnosis (2). An ileocecal resection (ICR) is the most common surgical procedure in CD (3). Despite an intestinal resection may induce disease remission and provide relief of CD symptoms, surgery is not curative and recurrence at the ileocolic anastomosis and/or in the neoterminal ileum is common (4-6).

Ileocolonoscopy is considered the golden standard for the diagnosis of postoperative recurrence in patients with CD (7). The Rutgeerts score (RS) was developed as an endoscopic scoring system to assess the severity of recurrence of inflammation at the ileocolic anastomosis and in the neoterminal ileum. The original RS stratifies the endoscopic severity into 5 groups (i0–i4) (8). High indices of the RS (≥i2) are associated with a higher risk of clinical recurrence and a re-resection when compared with a lower RS (i0–i1) (9). However, the prognostic value per index score of the RS is unknown.

The modified Rutgeerts score (mRS) was proposed to differentiate i2 into lesions confined to the anastomosis (i2a) versus lesions in the neoterminal ileum (i2b) and is currently used to assess the severity of postoperative endoscopic recurrence (10). The nature of anastomotic lesions (i2a) is unknown and may be related to a postischemic surgical phenomenon or related to staples, instead of CD recurrence (11). Several studies have reported conflicting clinical outcomes of anastomotic lesions on several measures of postoperative recurrence (clinical recurrence, surgical recurrence, and/or progression to [severe] endoscopic recurrence) (12–18). In a recently published individual participant data meta-analysis, no difference was observed between i2a and i2b lesions for clinical recurrence and/or a surgical reintervention (19). However, no adjustment for known risk factors was conducted for the latter outcome. In addition, progression to severe endoscopic recurrence was not assessed. Therefore, the initiation or optimization of medication after an endoscopic diagnosis of ulcerations at the ileocolic anastomosis remains a matter of debate.

In this cohort study, we assessed the prognostic value of the mRS (per index score), after correction for known clinical risk factors, to predict the risk of surgical and clinical recurrence, and progression to severe endoscopic recurrence after primary ICR in patients with CD.

## METHODS

### Participants and study design

Consecutive patients who underwent a primary ICR for the indication of CD between 2000 and 2019 were identified from a multicenter, retrospective database from 6 academic and 4 teaching hospitals in the Netherlands. All patients with CD (i) aged 16 years or older, (ii) who underwent ICR with restoration of the intestinal continuity, and (iii) who had ≥ 1 postoperative ileocolonoscopy assessed with the use of the mRS were included. Exclusion criteria were a permanent stoma, a re-resection before the first postoperative endoscopic assessment, prior intestinal resections, other indications for ICR (e.g., gastrointestinal malignancy), and/or absence of follow-up data.

### Outcomes

The primary outcome of this study was surgical recurrence (i.e., re-resection of the small bowel and/or colon) for CD recurrence during follow-up. Surgical recurrence within 3 months from primary ICR was considered as a re-resection due to postoperative complications and not considered as surgical recurrence. The secondary outcomes were (i) clinical recurrence defined as CD-related complaints with subsequent endoscopic recurrence (mRS ≥ i2b), surgical recurrence, radiologic recurrence (assessed by a local radiologist on ultrasonography, computed tomography, or magnetic resonance imaging), and/or therapeutic optimization (i.e., initiation of corticosteroids, immunomodulators, or biologicals for symptomatic disease) and (ii) progression to severe endoscopic recurrence (mRS ≥ i3) in patients with an index mRS i0–i2b.

### Data collection

Baseline and clinical data were retrieved from individual medical charts including demographics, surgical and disease characteristics, and prior medical treatment. The date of index ileocolonoscopy (i.e., first operative ileocolonoscopy) was set as start of the follow-up and time at risk of this study. The mRS at the first postoperative ileocolonoscopy (i.e., index mRS) was used to assess the outcomes. The mRS was graded separately by 4 trained physicians (S.B., J.A., E.B., and J.S.) based on available photographs and/or the endoscopy report for all patients. Follow-up time was defined as the interval between the index ileocolonoscopy (t0) and time to event. Patients were censored in case of the event was not observed (i.e., end of follow-up or lost to follow-up).

### Statistical analyses

Descriptive statistical analysis (frequency, percentage, mean, SD, median, and interquartile range [IQR]) was used to describe the research sample. Categorical variables were quoted as the number and percentage. Continuous variables were tested for normality using the Shapiro-Wilk test. Normal distributed variables were presented as mean and SD, while non-normal distributed variables were presented as median and IQR. Kaplan-Meier curves, with log-rank test for significance, were used to describe and compare survival probabilities between individual mRS.

Associations between index mRS and known clinical risk factors (according to the current guidelines) and the 3 time-to-event outcomes (surgical and clinical recurrence, and progression to severe endoscopic recurrence) were investigated using Cox proportional hazard models (7,20,21). The following variables were included for multivariable analysis: age at diagnosis, penetrating disease behavior at surgery (according to the Montreal classification), maintenance therapy during follow-up (i.e., continuation of postoperative prophylactic medication or start of medication within 6 weeks after index ileocolonoscopy with an antitumor necrosis factor agent [anti-TNF] and/or an immunomodulator), and time to index ileocolonoscopy (7,20,21). The models included a random effect for the study center to take potential correlation into account between patients treated in the same hospital.

Because severe endoscopic recurrence is not observed directly and only known to lie within the interval between the first ileocolonoscopy at which it was not yet present and the last ileocolonoscopy at which it was diagnosed, sensitivity analysis with interval censoring for severe endoscopic recurrence was performed. Analyses were performed in R version 4.1.3 (R Core Team 2022) with the help of the packages icenReg (version 2.0.15) and survival (22).

### Ethics

This study was performed in accordance with the Declaration of Helsinki and approved by the Medical Ethical Research Committee of the Erasmus University Medical Centre Rotterdam (MEC-2017-1151).

## RESULTS

### Baseline characteristics

A total of 652 patients with CD who underwent a primary ICR were included. Most of the patients were female (62.9%) with a mean age of 35.6 years (SD: 13.8) and a median disease duration of 3.1 years (IQR: 0.8–8.2) during ICR (Table 1). Disease localization was restricted to the ileum in 63.8% (n = 418) of patients, and 36.2% (n = 236) of patients had ileocolic disease at ICR. After primary ICR, postoperative prophylactic treatment was initiated in 36.7% (n = 239) of the patients and concerned immunomodulator monotherapy (61.1%, n = 146), anti-TNF monotherapy agent (21.8%, n = 52), combination therapy (immunomodulator and anti-TNF agent) (14.6%, n = 35), ustekinumab (2.1%, n = 5), and vedolizumab (0.4%, n = 1).

**Table 1.**
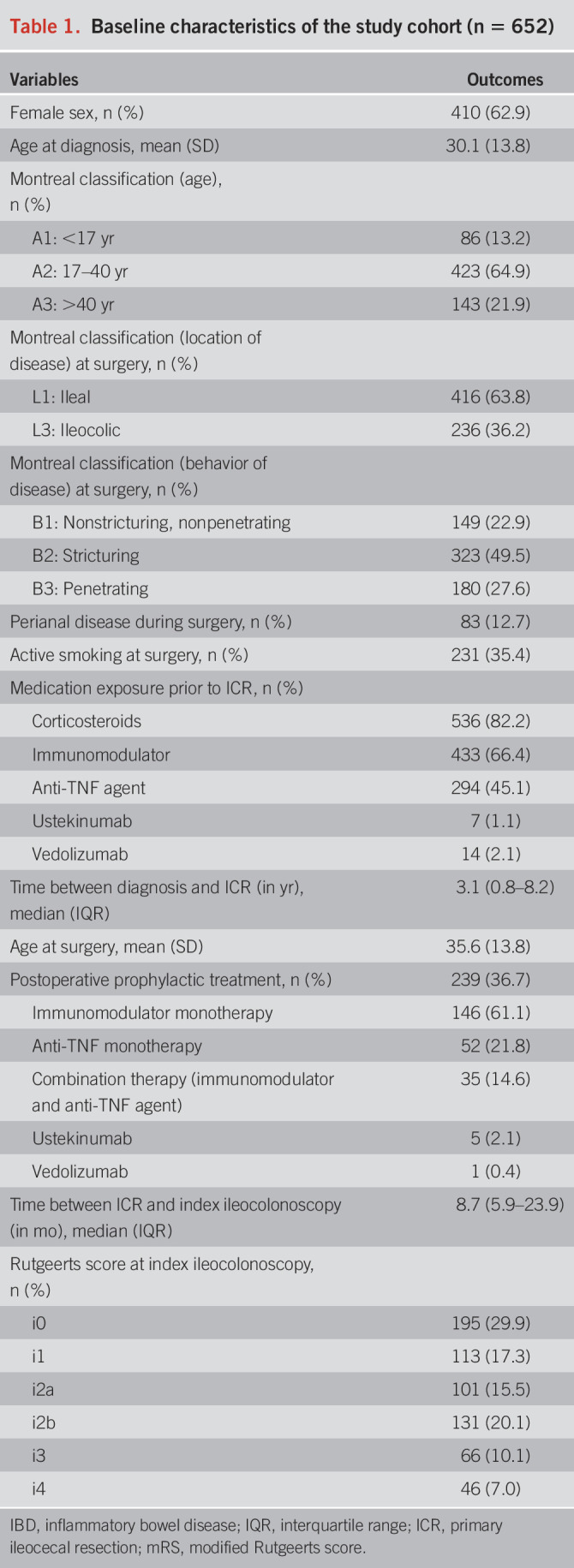
Baseline characteristics of the study cohort (n = 652)

Index ileocolonoscopy was performed at a median of 8.7 months (IQR: 5.9–23.9) after primary ICR. The mean follow-up period after index ileocolonoscopy was 6.4 years (SD: 4.6). The index mRS comprised i0 in 195 patients (29.9%), i1 in 113 patients (17.3%), i2a in 101 patients (15.5%), i2b in 131 patients (20.1%), i3 in 66 patients (10.1%), and i4 in 46 patients (7.0%). After the index ileocolonoscopy, maintenance therapy was initiated, within 6 weeks after ileocolonoscopy, in 14.4%, 14.2%, 30.7%, 43.5%, 50.0%, and 58.7% of patients with i0, i1, i2a, i2b, i3, and i4, respectively.

### Index modified Rutgeerts score and surgical recurrence

**Figure 1.**
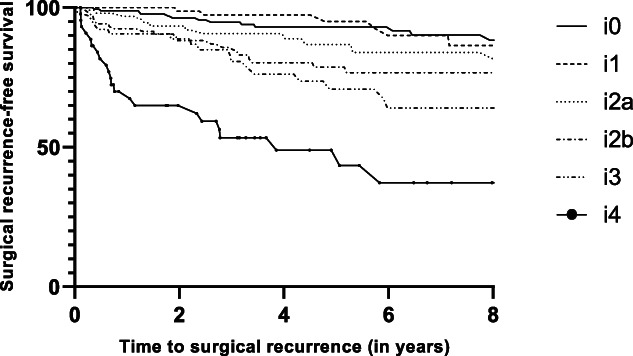
Kaplan-Meier curve of surgical recurrence-free survival (n = 652).

The overall surgical recurrence rate was 15.3% (n = 100) after a mean time to re-resection of 2.3 years (IQR: 0.6–4.5). During follow-up, surgical recurrence occurred in 7.7%, 5.3%, 12.9%, 19.1%, 28.8%, and 47.8% in patients with i0, i1, i2a, i2b, i3, and i4 (Figure 1). Surgical recurrence rates were not significantly higher in patients with an index mRS of i2b when compared with patients with an index mRS of i2a (28.8% vs 19.1%) (log-rank test, *P* = 0.16).

### Index modified Rutgeerts score and clinical recurrence

**Figure 2.**
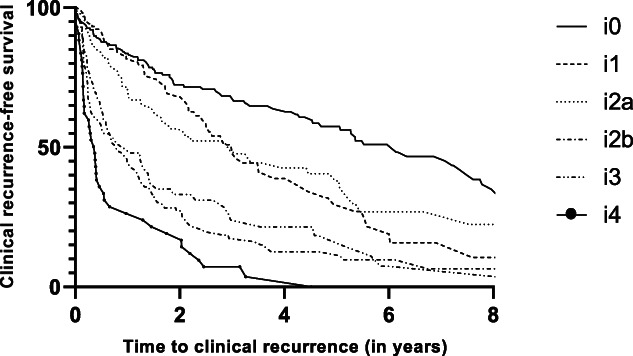
Kaplan-Meier curve of clinical recurrence-free survival (n = 626).

Six hundred twenty-six patients (96.0%) were eligible for the analysis on clinical recurrence. Clinical recurrence occurred in 63.1% (n = 412) of patients and was reported in 42.2%, 53.7%, 58.5%, 80.2%, 79.4%, and 95.3% in patients with i0, i1, i2a, i2b, i3, and i4 (Figure 2). Clinical recurrence rates were significantly higher in patients with an index mRS of i2b when compared with patients with an index mRS of i2a (80.2% vs 58.5%) (log-rank test, *P* < 0.001).

### Index modified Rutgeerts score and progression to severe endoscopic recurrence

**Figure 3.**
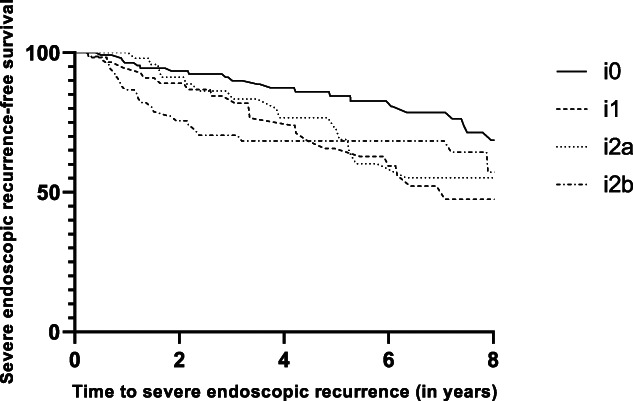
Kaplan-Meier curve of severe endoscopic recurrence-free survival (n = 304).

During follow-up, 55.9% of the patients (n = 304) (57.4% i0, 53.6% i1, 53.9% i2a, 57.3% i2b) with an index mRS i0–i2b underwent >1 postoperative ileocolonoscopy. In this subset of patients, progression to severe endoscopic recurrence (i3–i4) was reported in 27.7% of patients (n = 84). Progression to severe endoscopic recurrence rates occurred in 21.1% (i0), 33.9% (i1), 26.8% (i2a), and 33.3% (i2b) of patients (Figure 3). Severe endoscopic recurrence rates were not significantly higher in patients with an index mRS of i2b when compared with patients with an index mRS of i2a (33.3% vs 26.8%) (log-rank test, *P* = 0.47).

### Association of the modified Rutgeerts score with outcomes

After adjusting for the included clinical risk factors, an index mRS of i1 (adjusted hazard ratio [aHR] 0.7; 95% confidence interval [CI] 0.3–1.9]) and anastomotic lesions (i2a) (aHR 1.7; 95% CI 0.8–3.5) were not associated with surgical recurrence in multivariable analysis (Table 2). An index mRS of i2b (aHR 2.9; 95% CI 1.5–5.6), i3 (aHR 4.0; 95% CI 2.0–7.9), and i4 (aHR 8.0; 95% CI 4.0–16.0) were independently associated with surgical recurrence during follow-up. An increased time to index ileocolonoscopy was associated with surgical recurrence (aHR 1.1; 95% CI 1.0–1.2). No other associations were reported.

**Table 2.**
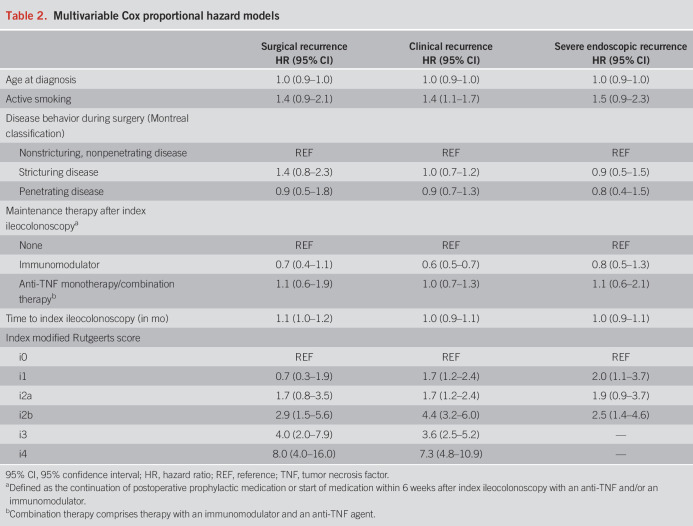
Multivariable Cox proportional hazard models

An index mRS of i1 (aHR 1.7; 95% CI 1.2–2.4), i2a (aHR 1.7; 95% CI 1.2–2.4), i2b (aHR 4.4; 95% CI 3.2–6.0), i3 (aHR 3.6; 95% CI 2.5–5.2), and i4 (aHR 7.3; 95% CI 4.8–10.9) were associated with clinical recurrence. Furthermore, active smoking at surgery (aHR 1.4; 95% CI 1.1–1.7) and maintenance therapy with an immunomodulator (aHR 0.6; 95% CI 0.5–0.7) were associated with clinical recurrence.

Concerning progression to severe endoscopic recurrence, an index mRS of i2a was not associated with progression to severe endoscopic recurrence (aHR 1.9; 95% CI 0.9–3.7). An index mRS of i1 and i2b were independently associated with progression to severe endoscopic recurrence (aHR 2.0; 95% CI 1.1–3.7 [i1]) (aHR 2.5; 95% CI 1.4–4.6 [i2b]). No clinical risk factors were associated with progression to severe endoscopic recurrence.

After interval censoring, sensitivity analysis showed no association of anastomotic lesions (i2a) with progression to severe endoscopic recurrence (aHR 1.8; 95% CI 0.9–3.8) (see Supplementary Table 1, http://links.lww.com/AJG/D48). In line with the earlier findings, an association for an index mRS of i2b, on progression to severe endoscopic recurrence, was observed in multivariable analysis (aHR 2.1; 95% CI 1.1–4.1).

## DISCUSSION

In this study, the increasing mRS corresponds closely with the risk of surgical and clinical recurrence in patients with CD after a primary ICR, but not with the risk of progression to severe endoscopic recurrence. In multivariable analysis, anastomotic lesions (i2a) were not associated with a re-resection, in contrast to an index mRS ≥ i2b. Similarly, anastomotic lesions were not associated with severe endoscopic recurrence, in contrast to mild lesions in the neoterminal ileum (index mRS of i1 or i2b). An index mRS ≥ i1 is associated with clinical recurrence. Tight monitoring to timely optimize medication seems indicated in patients with inflammation in the ileum (index mRS of i1 and ≥ i2b) to prevent progression to severe endoscopic recurrence and/or surgical recurrence. In patients with inflammation confined to the anastomosis, a more conservative approach seems appropriate.

Current American and European guidelines recommend escalation or initiation of medication in patients with an RS ≥ i2 (20,21). Refinement of these recommendations into mRS ≥ i2b seems indicated based on the findings of this study and previous observations on long-term outcomes of anastomotic lesions (14,17,18). The more indolent disease course in patients with anastomotic lesions when compared with ileal inflammation regarding progression to severe endoscopic lesions has also been shown in 2 retrospective multicenter studies (14,17). In addition, Hammoudi et al (18) reported a shorter clinical recurrence-free survival in patients with ileal lesions at index ileocolonoscopy when compared with patients with lesions confined to the anastomosis. These findings are in line with our results showing that an index mRS of i1 is associated with both clinical recurrence and progression to severe endoscopic recurrence, whereas an index mRS of i2a is merely associated with clinical recurrence. These outcomes may be explained by a distinct pathological mechanism of anastomotic lesions when compared with ileal lesions, in which the role of ischemia is debated (18,23). A recent published meta-analysis with individual patient data reported no difference was observed between i2a and i2b lesions on the outcomes of clinical recurrence and/or a surgical reintervention (19). However, the analyses for a surgical reintervention were not corrected for known risk factors associated with recurrence. In this study, after adjusting for known clinical risk factors, an index mRS ≥ i2b was found to be independently associated with surgical recurrence and progression to severe endoscopic recurrence, which supports the recommendation to consider therapy optimization in patients with an index mRS of ≥ i2b after primary ICR.

Despite the lack of a statistically significant association between anastomotic lesions and surgical recurrence and progression to severe endoscopic recurrence, the risks for both outcomes were still as high as 12.7% and 26.8% during follow-up. Further research to identify risk factors and/or biomarkers for postoperative recurrence is warranted to appropriately manage patients with anastomotic lesions. The need for more accurate biomarkers seems underscored by the lack of association between clinical risk factors, except for active smoking and maintenance therapy with an immunomodulator with clinical recurrence and long-term outcomes in multivariable analysis in this study.

Recently, a new endoscopic scoring system has been proposed in which endoscopic scoring should be adapted to the anastomotic technique (24). The (m)RS has been developed for the assessment of an end-to-end anastomosis. In the modern era, a wide lumen stapled side-to-end or side-to-side anastomosis has been preferred over the end-to-end anastomosis to prevent anastomotic leakage, fecal stasis, and stenosis of the anastomosis. When the (m)RS is applied to endoscopically assess these anastomotic techniques, anatomic locations such as the ileal blind loop and ileal body are disregarded (24,25). Prospective analysis of inflammation at these locations and subsequent refinement of the endoscopic score is awaited.

Our study was the first to assess the predictive value of the mRS on long-term outcomes in postoperative patients with CD. Despite the consideration of objective outcome measures in a large population (from both academic and nonacademic hospitals) of patients who underwent a primary ICR with long-term follow-up, limitations of this study need to be taken into consideration. First, because the indication of subsequent ileocolonoscopies could not be assessed, due to the retrospective design, confounding by indication may be present. Second, because our study concerns a wide period, several changes of postoperative management may have influenced the outcomes including improved access to endoscopy, development of strict and noninvasive monitoring, and medication strategies. This study design did not allow to correct for all these potential confounding factors. Regarding the changes in the postoperative endoscopic strategy, a substantial number of patients (40%) did not undergo an index ileocolonoscopy within 1 year postoperatively, which is recommended by the current guidelines (7,20,21). To adjust for potential confounding, we have included time to index ileocolonoscopy in the multivariable analysis. Finally, perianal fistulizing disease, plexitis, and/or granulomas in the resection specimen are considered risk factors of postoperative recurrence in current guidelines (7,20,21). Due to the restriction of number of variables that could be included in multivariable analysis, the findings are not corrected for the presence of perianal fistulas. In addition, standardized data on the presence of plexitis and/or granulomas in the resection specimen were unavailable in the pathology reports.

In conclusion, the increasing mRS at index ileocolonoscopy corresponds closely with the risk of surgical and clinical recurrence after primary ICR. Anastomotic lesions (i2a) are not associated with surgical recurrence and progression to severe endoscopic recurrence, in contrast to lesions in the neoterminal ileum (≥i2b). An index mRS ≥ i1 is associated with clinical recurrence. In addition, i1 lesions are associated with progression to severe endoscopic recurrence. These results support conservative management and no need for escalation of therapy in patients with anastomotic lesions and tight monitoring of disease activity and treatment optimization in patients with ileal lesions.

## CONFLICTS OF INTEREST

**Guarantor of the article:** Annemarie C. de Vries, MD, PhD.

**Specific author contributions:** M.T.J.B., S.t.B.H., A.C.d.V.: conception and design: M.T.J.B, S.t.B.H., F.H., A.G.L.B., G.D., M.J.R.-C., N.K.H.d.B., L.P.S.S., A.E.v.d.M.d.-J., R.L.W., O.v.R., C.v.d.W., A.C.d.V.: acquisition and interpretation of data: M.T.J.B., S.t.B.H., N.S.E., A.C.d.V.: analysis of data. All authors have participated in drafting or critically revising the manuscript and gave their final approval of the current version.

**Financial support:** None to report.

**Potential competing interests:** M.T.J.B. has served as speaker for Abbvie outside the submitted work. A.G.L.B. has served on the advisory board of Takeda, Abbvie, and Janssen outside the submitted work. Gerard Dijkstra has received a grant from Royal *DSM* and speaker fees from: Abbvie, Janssen-Cilag, Takeda, and Pfizer outside the submitted work. N.K.H.d.B. has served as a speaker for AbbVie and MSD and has served as consultant and principal investigator for TEVA Pharma BV and Takeda. He has received an (unrestricted) research grant from Dr. Falk, TEVA Pharma BV, MLDS, and Takeda, all outside the submitted work. F.H. has served on advisory boards or as speaker for Abbvie, Janssen-Cilag, MSD, Takeda, Celltrion, Teva, Sandoz, and Dr Falk, and has received funding (Grants/Honoraria) from Dr. Falk, Janssen-Cilag, Abbvie, and Takeda and consulting fees from Celgene and Janssen-Cilag, all outside the submitted work. L.P.S.S. has served as a speaker and received research support from Takeda, outside the submitted work. A.E.v.d.M.d.J. reports presentation fee from Janssen and has served on the advisory board of Takeda and Galapagos, outside the submitted work. R.L.W. has served on the advisory board and as invited speaker for Janssen, Pfizer, Takeda, and Galapagos, outside the submitted work. C.J.v.d.W. received grants and or fee for advisory boards and presentations from Pfizer, Abbvie, Celltrion, Falk Benelux, Takeda, Janssen, and Ferring, outside the submitted work. O.v.R. has served as invited speaker for Janssen-Cilag and has received nonfinancial support from Takeda, outside the submitted work. A.C.d.V. has served on advisory boards for Takeda, Janssen, Bristol Myers Squibb, Abbvie, Pfizer, and Galapagos and has received unrestricted research grants from Takeda, Janssen, and Pfizer, outside the submitted work. All other authors report no conflicts of interests.

**Ethical approval statement:** The RAP-CD study was approved by the Medical Ethics Review Committee of the Erasmus Medical Center (METC-2017-482). The study protocol conformed to the ethical guidelines of the 1975 Declaration of Helsinki.

**Study Highlights**

**WHAT IS KNOWN**

✓ The modified Rutgeerts score (mRS) is used for the assessment of postoperative recurrence in Crohn's disease.
✓ The prognostic value of the mRS for long-term outcomes needs to be further elucidated, especially the impact of anastomotic lesions needs clarification.

**WHAT IS NEW HERE**

✓ The increasing mRS corresponds closely with the risk of surgical and clinical recurrence.
✓ Moderate-to-severe lesions (≥i2b) are associated with surgical recurrence and mild-to-moderate lesions are associated with progression to severe endoscopic recurrence (i1 and i2b).
✓ An mRS ≥ i1 is associated with clinical recurrence.

## Supplementary Material
