# Supplementary material for: Prognostic Value of the Modified Rutgeerts Score for Long-Term Outcomes After Primary Ileocecal Resection in Crohn's Disease
Source: Am J Gastroenterol. 2023 Nov 1;119(2):306–12. doi: 10.14309/ajg.0000000000002509 (PMC10833187; doi:10.14309/ajg.0000000000002509)
Supplement: Supplementary file 1 [file acg-119-306-s001.docx]

**Supplementary Tables**

**Supplementary Table 1.** Sensitivity analysis with interval censoring for severe endoscopic recurrence

|  | **Severe endoscopic recurrence** |
| --- | --- |
|  | HR (95% CI) |
| Age at diagnosis | 1.0 (0.9 – 1.0) |
| Active smoking | 1.6 (0.9 – 2.5) |
| Disease behaviour at time of surgery (Montreal classification)  *Non-stricturing, non-penetrating disease*  *Stricturing disease*  *Penetrating disease* | REF  1.0 (0.5 – 1.8)  0.9 (0.5 – 1.7) |
| Maintenance therapy after index ileocolonoscopy  *None*  *Immunmodulator*  *Anti-TNF monotherapy/combination therapy*^¥^ | REF  0.8 (0.4 – 1.5)  1.0 (0.5 – 2.1) |
| Time to index ileocolonoscopy (in months) | 0.8 (0.7 – 0.9) |
| Index modified Rutgeerts score  *i0*  *i1*  *i2a*  *i2b*  *i3*  *i4* | REF  1.7 (0.9 – 3.4)  1.8 (0.9 – 3.8)  2.5 (1.1 – 4.1)  -  - |

**Abbreviations.** HR = hazard ratio; 95% CI = 95% confidence interval; REF = reference; TNF = tumour necrosis factor.

^¥^ Combination therapy comprises therapy with an immunomodulator and an anti-TNF agent.
